# Assessing the Premedication Properties of Sublingual Melatonin in Young Women Undergoing Cesarean Section With Spinal Anesthesia: A Double-Blind Randomized Study

**DOI:** 10.7759/cureus.59710

**Published:** 2024-05-06

**Authors:** Hussein J Alkhfaji, Hussein A Hussein, Majid F Mutar, Mohamed Kahloul

**Affiliations:** 1 Department of Anaesthesiology and Intensive Care, Ibn El Jazzar Medical Faculty of Sousse, Sousse, TUN; 2 Department of Anesthesia, College of Health and Medical Technologies, Al-Ayen Iraqi University, Nasiriyah, IRQ; 3 Department of Anaesthesiology and Intensive Care, Teaching Hospital of Sahloul, Ibn El Jazzar Medical Faculty of Sousse, Sousse, TUN

**Keywords:** premedication, sedation, preoperative anxiety, spinal anesthesia, melatonin

## Abstract

Introduction: Preoperative anxiety can negatively impact patient outcomes by influencing the intraoperative requirements for anesthetics and analgesics, increasing postoperative pain intensity, and augmenting the need for analgesia. Moreover, it may contribute to higher rates of postoperative morbidity and mortality following certain types of surgery. This study investigates the anxiolytic and sedative properties of sublingual melatonin as a premedication agent in young females undergoing cesarean section under spinal anesthesia.

Methods: A double-blind, randomized, placebo-controlled trial was conducted in Nasiriyah, Iraq. Eighty females were included, 40 in each group, based on specific inclusion and exclusion criteria. Premedication was administered in the morning, 60 minutes before the procedure. In the melatonin group (M), patients received 10 mg of sublingual melatonin, while the placebo group (P) received placebo premedication. Anxiety and sedation levels were evaluated three times: before taking premedication, five minutes before the insertion of the spinal needle, and one hour postoperatively, using the visual analog scale and Richmond Sedation Scale.

Results: The results show a highly significant P-value regarding anxiety levels between the M Group and P Group (p-value < 0.001). There was a significant difference in the median sedation scores between the studied groups at pre-spinal insertion and postoperatively (p-value < 0.001). The mean heart rate in the M Group was significantly lower than in the P Group (p-value = 0.0019). Significant differences were noted in systolic and diastolic blood pressures between the two groups, measured five minutes before and after spinal needle insertion (p-value < 0.001).

Conclusion: These findings contribute to understanding the impact of sublingual melatonin as an anxiolytic and sedative premedication agent on patients undergoing elective cesarean sections under spinal anesthesia. Further research is warranted to fully elucidate the benefits and implications of melatonin administration in such procedures.

## Introduction

Anxiety, discomfort, and nervousness can intensify due to heightened levels of fear, tension, and apprehension; especially in the preoperative time [1]. Females undergoing cesarean section often experience anxiety stemming from the surgical procedure itself or fear associated with the operating room environment. Additionally, the autonomic response triggered by the surgical setting can exacerbate this anxiety. Uterine blood flow can get compressed by these stress responses leading to fetal distress [2,3]. Apart from anxiety, spinal anesthesia commonly triggers autonomic sympathetic blockade, causing a decrease in systemic vascular resistance and subsequent hypotension. This effect can be mitigated or addressed through various pharmacological and non-pharmacological interventions, including left uterine displacement, preloading or co-loading with crystalloids or colloids fluids, and the administration of vasopressors such as ephedrine and phenylephrine [4].

Premedication involves the administration of medications before surgery with the aim of reducing or alleviating patient anxiety prior to, during, and after the surgical procedure [5]. Various medications have been utilized to alleviate anxiety prior to surgery over the last three decades [6,7]. Administering midazolam or mirtazapine preoperatively for females undergoing cesarean section has been shown to reduce the level of anxiety. However, it's worth noting that both midazolam and mirtazapine may be associated with some unfavorable side effects like an increase in the incidence of postoperative nausea and vomiting (PONV) [2,8,9].

Some previous and recent studies have suggested that the use of melatonin before cesarean surgery, has a role in reducing the oxidative stress in septicemic newborns, enhancing the baby outcomes, and reducing the possibility of oxidative stress incidence or damaging of blood supply of asphyxiated newborns [3,9,10].

The aim of this study was to assess the effectiveness of sublingual melatonin as a premedication agent in inducing sedation and alleviating preoperative anxiety among young females undergoing elective cesarean surgery under spinal anesthesia.

## Materials and methods

This was a randomized double-blind study conducted in the obstetric surgery department at the Bint Al-Huda Teaching Hospital in Nasiriyah, Iraq, from August 2022 to January 2023. The study was approved by the Thi-Qar Directorate Ethical Committee, Ministry of Health, Iraq (approval number: 37/2021) and registered in the Iranian Registry of Clinical Trials (ID IRCT20230809059105N1). 

Inclusion and exclusion criteria

Female patients undergoing cesarean section who agreed to participate in the study were included in the study provided they met the following criteria: 18-45 years of age, classified as American Society of Anesthesiologists (ASA) II, gestational period exceeding 37 weeks, intact membranes, having a singleton pregnancy, and scheduled surgical operation.

The study excluded patients classified ≥ ASA III, individuals with documented drug allergies to melatonin or any other study medications, patients contraindicated for regional anesthesia such as spinal deformity or infection. The inability of patients to respond or demonstrate awareness to posed questions, chronic anemia (hemoglobin levels below 8 g%), and a history of mental or neurological disorders also served as exclusion criteria. Furthermore, patients with substance addiction that could interfere with study outcomes, the presence of congenital malformations in the fetus, and patients who did not give consent for participation were excluded.

Sample size calculation

The determination of the sample size was based on the anticipated changes in hemodynamics. Considering the relevant metrics, the effect size was calculated to be 0.45 with a standard deviation of 0.71. To maintain a minimum power of 80% and a maximum type I error of 0.05, a minimum sample size of 80 patients was calculated. This sample was separated into two groups, with each group including 40 patients.

Randomization and premedication

**Figure 1:**
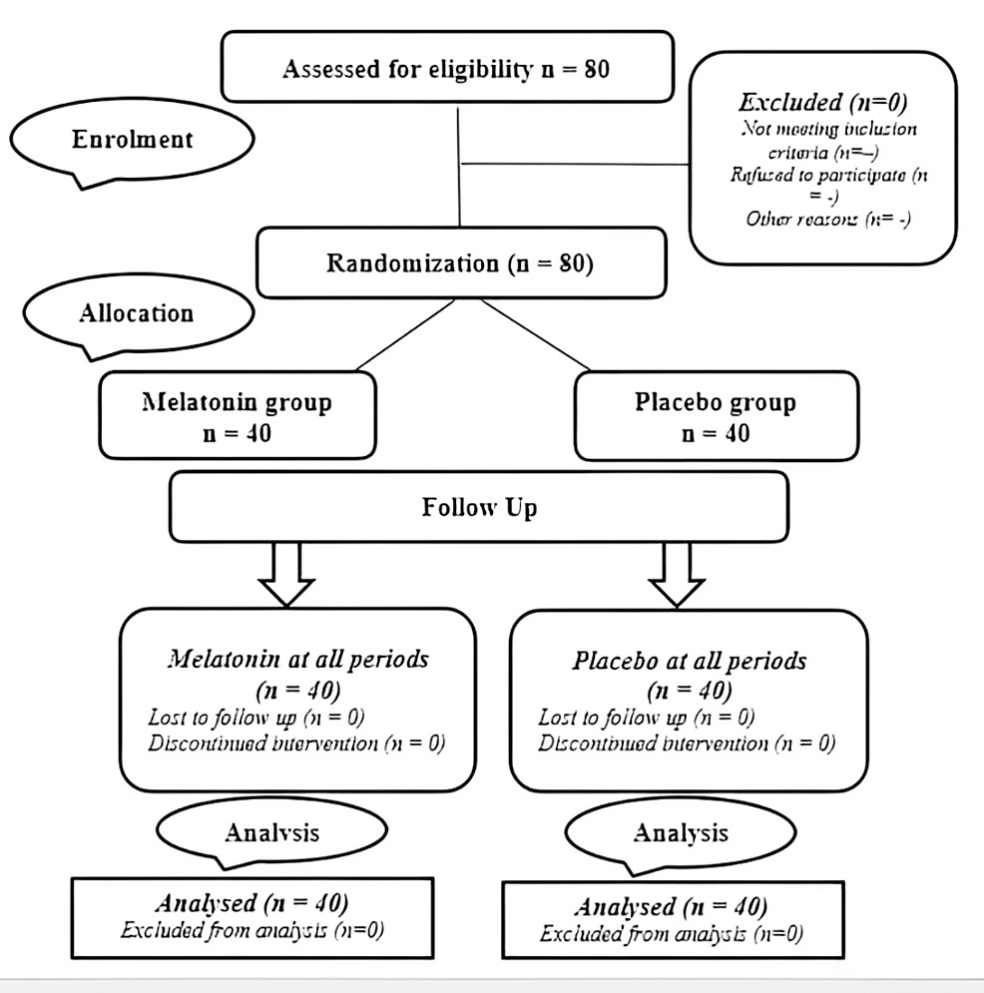
CONSORT flowchart CONSORT: Consolidated Standards of Reporting Trials

Enrolled patients were randomly assigned to one of two groups, each comprising 40 patients (Figure 1). The first group, referred to as the melatonin group (M), received melatonin as premedication, while the second group, designated as the placebo group (P), received a placebo. In both groups, the premedication was administered at the ward by a nurse who was not enrolled in the study. In the M Group, the patients received 10 mg of sublingual melatonin in the morning, 60 minutes before getting into the operating room. The patients in the P Group received oral placebo premedication at the same time as the first group.

Data collection

The levels of both anxiety and sedation were evaluated completely by the anesthesiologist before the administration of premedication, five minutes before the spinal needle insertion (SNI), and one hour postoperatively depending on the visual analog scale (VAS) and Richmond Sedation Scale. In the VAS, a 10 cm ruler was used to evaluate the anxiety level, in which 0 indicated no anxiety and 10 indicated a severe anxiety level.

Perioperative monitoring

All patients underwent standard monitoring, including electrocardiography (EKG), non-invasive blood pressure (NIBP), and pulse oximetry with a patient monitor (Nihon Kohden Corporation, Tokyo, Japan). Blood pressure, including systolic blood pressure (SBP), diastolic blood pressure (DBP), and mean arterial pressure (MAP), was continuously monitored at various intervals: preoperatively, intraoperatively at five minutes, 10 minutes, and 20 minutes, and postoperatively at 15 minutes, 60 minutes, and 120 minutes.

Spinal procedure

Before administering the subarachnoid block, a preload lactated ringer solution was infused into all patients at a rate of 5 ml/kg. For the spinal anesthesia, a Quincke-type needle with a 25-gauge was introduced intrathecally after the aseptic technique into the L4-5 interspace with the midline approach while the patient was in a sitting position. A 10 mg of bupivacaine was used for this purpose. Oxytocin was administered shortly after the neonate's delivery via intravenous infusion over 15 minutes, dissolved in 0.5 L of Ringer lactate solution. The dosage was given based on the surgeon's assessment of uterine tone. Following the end of surgery, patients were transferred to the ward with a patient-controlled analgesia (PCA) system to relieve postoperative pain. A 100 cc of normal saline with 25 mg of morphine was infused by PCA pump. The PCA pump parameters included a bolus size of 0.5 cu cm and a lockout interval of 15 minutes.

Statistical analysis

The obtained data from the study samples was revised, coded, and tabulated by using the IBM SPSS Statistics for Windows, Version 25.0 (Released 2017; IBM Corp., Armonk, New York, United States). Accordingly, data were presented for suitable analysis according to the type of data relevant to each parameter. A p-value lower than 0.05 was considered significant.

## Results

The study included 80 patients randomly divided into two groups: the M Group (n=40) and the P Group (n=40). Upon statistical analysis, no significant differences in sociodemographic characteristics were found between the groups. Specifically, there were no notable discrepancies in mean age, weight, height, and BMI between the study groups, with p-values of 0.378, 0.144, 0.062, and 0.726, respectively (Table 1). 

**Table 1:** Comparison between melatonin and placebo groups with regard to demographic data M Group: melatonin group; P Group: placebo group

	M Group (n =40)	P Group (n =40)	p-value
Age (years), mean ± SD	28.85 ± 7.46	30.20 ± 6.08	0.378
Weight (kg), mean ± SD	77.60 ± 10.90	74.48 ± 7.76	0.144
Height (cm), mean ± SD	165.03 ± 7.91	162.30 ± 4.43	0.062
BMI (kg/m^2^), mean ± SD.	28.50 ± 3.37	28.27 ± 2.64	0.726

VAS scores revealed a significant difference in anxiety levels between the two groups. Specifically, the P group exhibited higher anxiety levels both before premedication and one hour postoperatively compared to the M group (p-value < 0.001) (Table 2).

**Table 2:** Comparison between melatonin and placebo groups with regard to anxiety level M Group: melatonin group; P Group: placebo group; SNI: spinal needle insertion; VAS: visual analog scale ^**^ p-value is highly significant; Mild VAS: 2-3; Moderate VAS: 4-5; Severe VAS: 6-7; Very severe VAS: 8-9

	M Group	P Group	p-value
Preoperative Anxiety			0.004
Mild VAS	7	0	
Moderate VAS	31	29	
Severe VAS	2	10	
Very severe VAS	0	1	
Five minutes before SNI			<0.001**
Mild VAS	23	2	
Moderate VAS	17	36	
Severe VAS	0	2	
Very severe VAS	0	0	
Postoperative Anxiety			<0.001**
Mild VAS	36	13	
Moderate VAS	4	27	
Severe VAS	0	0	
Very severe	0	0	

There was no significant difference in sedation scores between the two groups with regard to the sedation level before the administration of the morning dose of premedication. However, there was a statistically significant difference in the median sedation scores between the studied groups at pre-spinal insertion and postoperatively. The p-values in those two time-points were less than 0.001 (Table 3).

**Table 3:** Comparison between the melatonin and placebo groups with regard to Richmond sedation score M Group: melatonin group; P Group: placebo group; SE: standard error ^**^ p-value is highly significant

	M Group	P Group	p-value
Sedation before the 2^nd^ dose of premedication, mean ± SE	-0.16 ± 0.07	-0.24 ± 0.12	0.110
Sedation level before spinal needle insertion, mean ± SE	-0.76 ± 0.17	0.0 ± 0.0	<0.001**
Postoperative sedation level, mean ± SE	-2.08 ± 0.08	0.0 ± 0.0	<0.001**

There were no significant differences observed in baseline hemodynamic variables between the two groups. However, notable variations were noted in heart rate (HR) readings, with a statistically significant difference observed. The mean HR in the M Group was significantly lower than in the P Group. Similarly, no significant differences were observed in SBP at baseline; however, statistically significant differences were noted in DBP between the two groups across all time periods (Table 4).

**Table 4:** Comparison between the melatonin and placebo groups with regard to hemodynamic readings M Group: melatonin group; P Group: placebo group; SNI: spinal needle insertion ^*^ p-value is significant; ^**^ p-value is highly significant

Hemodynamics (Mean ± SD)	M Group	P Group	p-value
Baseline Readings, mean ± SE			
Heart rate	77.0 ± 9.64	78.32 ± 14.12	0.267
Systolic blood pressure	123.35 ± 17.75	127.05 ± 15.96	0.330
Diastolic blood pressure	85.40 ± 4.98	86.20 ± 2.61	0.131
Five Minutes Before SNI, mean ± SE			
Heart rate	81.88 ± 9.67	74.12 ± 6.36	0.0019*
Systolic blood pressure	136.33 ± 13.38	126.30 ± 16.92	0.004*
Diastolic blood pressure	76.40 ± 12.33	87.36 ± 5.86	<0.001**
Five Minutes After SNI, mean ± SE			
Heart rate	87.92 ± 16.53	100.6 ± 6.69	0.002*
Systolic blood pressure	139.1 ± 13.90	114.6 ± 9.08	<0.001**
Diastolic blood pressure	89.44 ± 8.07	73.44 ± 9.03	<0.001**
10 Minutes After SNI, mean ± SE			
Heart rate	79.32 ± 9.86	78.60 ± 6.52	0.133
Systolic blood pressure	132.3 ± 5.94	91.0 ± 20.36	<0.001**
Diastolic blood pressure	80.16 ± 8.92	85.68 ± 4.73	0.002*

## Discussion

In the current study, we initially screened 92 patients for eligibility. Among them, five were excluded due to not meeting the inclusion criteria, and seven declined to participate. Consequently, a total of 80 patients were enrolled and allocated into two groups: the M group (n=40) and the P group (n=40). There was no significant difference between the groups with regard to age, weight, height, and BMI.

Based on the data on anxiety and sedation from our study, we have concluded that premedicating patients with 10 mg of sublingual melatonin significantly reduces anxiety levels and provides notable sedative effects compared to placebo premedication. Additionally, this dose of sublingual melatonin proved effective in enhancing analgesia and reducing the incidence of spinal-related issues in patients undergoing cesarean section under spinal anesthesia. Our findings regarding the analgesic effect of melatonin align with earlier studies, further supporting the efficacy of melatonin as a premedication agent [3,9,11-13]. The results of studies by Khare et al. [14] and Lotfy and Ayaad [15] align with that of the current study. Khare et al. discovered that premedication with oral melatonin (6 mg) serves as an effective alternative to alprazolam, offering improved anxiolysis, less sedation, and maintenance of cognitive and psychomotor function [14]. Furthermore, a systemic literature review by Campbell et al. revealed that perioperative melatonin, administered in daily doses of 2-8 mg for one to nine days, starting on the evening before or the day of surgery, reduced the incidence of postoperative complications such as anxiety and delirium following major surgeries [16].

Melatonin's role in regulating the circadian rhythm may contribute to its sedative and hypnotic effects [17]. Simultaneously, melatonin may exert its effects by modulating the inhibitory receptors named gamma-aminobutyric acid (GABAA) receptors by its interaction with melatonin receptors (MT1 and MT2) [18]. By affecting the GABAA receptor through the G protein-coupled pathway, melatonin has the ability to enhance the binding of GABA to the GABAA receptor. This mechanism has a similar pathway as other anesthetic drugs like propofol and benzodiazepines [13]. Melatonin induces its effects by stimulating GABAergic activity. This interaction between melatonin and GABAergic pathways seems to underlie neuropsychological effects, such as the hypnotic activity of melatonin via GABAA receptors. This activity is blocked by GABAergic antagonists. These alterations induced by melatonin in neurosignaling pathways may contribute to its anxiolytic effects [19].

The study results showed that the anxiety level in the M Group was less than that in the P Group at all time points of anxiety assessment, especially at the time of SNI and after ending the procedure (p < 0.001). The findings from the study conducted by Javaherforooshzadeh et al. in 2018 indicated that both the gabapentin and melatonin groups experienced significantly lower levels of pain and anxiety compared to the placebo group [20]. Additionally, higher satisfaction levels were reported among participants in the gabapentin and melatonin groups. A qualitative systematic review conducted by Yousaf et al. demonstrated that premedication melatonin has an effective role in lowering anxiety [13]. Melatonin also demonstrated a significant reduction in the occurrence of depressive symptoms among women with breast cancer in the three-month post-surgery period [21].

The effect of melatonin on intraoperative blood pressure has been investigated in various studies, but the results have been inconsistent. While some studies have shown that melatonin administration can lead to a decrease in blood pressure, especially in hypertensive individuals, other studies have reported differing outcomes [22]. The decrease in blood pressure observed may be attributed to melatonin's interaction with the autonomic nervous system, and its regulatory effects on vascular tone [23]. Current evidence, although limited, suggests that melatonin can stabilize and control systemic blood pressure within normal limits, aiding in its regulation [24]. Further investigation is required to gain a comprehensive understanding of the effects of melatonin on postoperative blood pressure. It's crucial to recognize that individual responses to melatonin may vary due to factors such as baseline blood pressure, existing health conditions, and concurrent medication use [25]. Moreover, the concurrent administration of other medications during surgery and the subsequent recovery period may interact with melatonin, potentially influencing blood pressure [26].

## Conclusions

Based on the findings, it can be concluded that the use of sublingual premedication of melatonin for females undergoing cesarean section yields favorable outcomes across the variables examined in this study. Sublingual melatonin at a dosage of 10 mg appears to be effective in alleviating preoperative anxiety in the study population. Additionally, the results indicate the significant efficacy of melatonin in inducing sedation during and after the procedure.
